# Cross-Modal Object Detection Based on Content-Guided Feature Fusion and Self-Calibration

**DOI:** 10.3390/s25113392

**Published:** 2025-05-28

**Authors:** Liyang Ning, Xuxun Liu, Luoyu Zhou, Xueyu Zou

**Affiliations:** 1 School of Electronic Information and Electrical Engineering, Yangtze University, Jingzhou 434023, China; 2022710611@yangtzeu.edu.cn (L.N.); liuxuxun@scut.edu.cn (X.L.); luoyuzh@yangtzeu.edu.cn (L.Z.); 2 College of Electronic and Information Engineering, South China University of Technology, Guangzhou 510641, China

**Keywords:** object detection, cross-modal, YOLOv8, transformer, self-calibration

## Abstract

Traditional transformers suffer from limitations in local attention, which can result in inadequate feature representation and reduced detection accuracy in cross-modal object detection tasks. Additionally, deep features are prone to degradation through multiple convolutional layers, leading to the loss of detailed information. To address these issues, we propose a dual-backbone cross-modal object detection model based on YOLOv8n. First, we introduce a parallel network in the backbone to enable the model to process information from different modalities simultaneously. Second, we design a content-guided fusion module (CGF) in the feature extraction network, leveraging both transformer and convolution operations to capture global and local information, thereby enhancing the model’s ability to focus on detailed object features. Finally, we propose an adaptive calibration fusion module (ACF) in the neck to fuse shallow and deep features, supplementing fine-grained details and improving the model’s detection capability in complex environments. Experimental results show that on the LLVIP dataset, our model achieves mAP50 of 96.4 and mAP95 of 63.8; on the M3FD dataset, it achieves mAP50 of 83.7 and mAP95 of 56.6. Our model outperforms baseline models and other state-of-the-art methods in detection accuracy, demonstrating robust performance for cross-modal object detection tasks across various environments.

## 1. Introduction

In recent years, object detection has become a core task in computer vision. However, in complex scenarios (such as at night, in heavy fog, or with occluded objects), detection methods based on a single modality (e.g., visible light images) often suffer from performance degradation due to environmental changes, making them unsuitable for tasks in such challenging environments. To address this issue, visible light-thermal (RGB-T) object detection has gradually become a research hotspot. Here, RGB refers specifically to color images captured by visible light cameras, which provide rich texture and color information, while T (thermal) images are captured by thermal cameras, utilizing thermal radiation information from objects, which allows them to work effectively even in complete darkness. The combination of these two modalities significantly improves the robustness and reliability of detection algorithms through their complementary characteristics. However, designing efficient cross-modal fusion mechanisms to fully mine the information from both visible light and thermal images remains a key challenge.

In existing work, most researchers have implemented multimodal information fusion based on convolutional neural networks (CNNs). Due to their powerful local perception ability and translation invariance, CNNs are effective in capturing local details, such as textures, edges, and shapes, while maintaining good detection performance even with changes in the object’s position. Zhang et al. [1] encoded the correlation between different modalities and adaptively fused features. Wolpert et al. [2] directly localized objects through key point prediction, improving small object detection capabilities. Zhang et al. [3] introduced a guided attention mechanism for dynamic adjustment of modality weights. Cao et al. [4] used a channel feature fusion module to integrate features from different modalities based on lighting conditions. However, due to the limitation of the receptive field, CNNs struggle to focus on global features, leading to insufficient capture of long-range dependencies. To address this issue, some researchers have recently introduced transformers [5] into the RGB-T object detection field. Transformers model global dependencies through self-attention mechanisms, partially compensating for CNNs’ inability to capture global features. However, classic transformers, which rely on global modeling, face difficulties in effectively capturing local features, such as texture and edge details. The lack of these local features can adversely affect the fusion of RGB and thermal images. Moreover, frequent convolution operations in neural networks can lead to feature degradation, which should not be overlooked as it affects the final detection performance.

To address the aforementioned challenges, we propose a dual-backbone cross-modal object detection model, named Multimodal Fusion YOLO (MFYOLO). Built upon the YOLOv8 framework, MFYOLO introduces the following innovative modules:

(1) Content-Guided Fusion Module (CGF): By embedding convolutional modules within the transformer architecture, CGF utilizes multi-head attention to capture global features while leveraging convolutional operations for feature processing. This enables joint modeling of global receptive fields and local detail features, effectively mitigating the traditional transformer’s shortcomings in local feature representation.
(2) Adaptive Calibration Fusion Module (ACF): By fusing shallow and deep features, ACF adaptively optimizes feature representation, significantly alleviating feature degradation caused by deep convolutional processes, particularly the loss of detailed information.

The main contributions of this paper are summarized as follows:

(1) The backbone was improved by adding a parallel network. The proposed CGF was embedded to enhance the model’s capability to represent both global and local features. Additionally, it facilitated the interaction between different modal features.
(2) At the end of the neck, the ACF was incorporated. Shallow-layer information was utilized to calibrate deep feature representations, thereby improving the model’s detection performance in complex environments.
(3) The proposed method was validated on multiple datasets. Experimental results demonstrated that it significantly outperformed existing state-of-the-art methods in terms of detection accuracy and robustness.

## 2. Related Work

In recent years, research on multispectral object detection has made continuous progress, especially in the field of RGB-T object detection. Liu et al. [6] designed four different fusion architectures (early fusion, late fusion, mid-level fusion, and score fusion), integrating two modality branches at different stages of deep neural networks, and revealed that the mid-level fusion model provides the best performance. Hou et al. [7] proposed a CNN-based pixel-level image fusion method, which automatically extracts features from images using deep learning without relying on manually designed features. Zhou et al. [8] leveraged the characteristics of differential amplifiers to understand the consistency and differences between different modalities through differential modal perception. Zheng et al. [9] introduced two novel Gate Fusion Units (GFU) to study the combination of feature maps generated by two SSD [10] middle layers. Park et al. [11] designed a CNN architecture that analyzes the detection probability of each modality and selectively uses channel-weighted fusion to improve detection performance. Zhang et al. [12] proposed a new cyclic fusion-refinement module, which improved multispectral feature fusion while balancing feature complementarity and consistency. To capture discriminative target features in multispectral images, MSDS-RCNN [13] jointly optimized pedestrian detection and semantic segmentation tasks. Li et al. [14] built a system called Illumination-Aware Network (IAN), which uses gate units to predict illumination coefficients for RGB images and then fuses the outputs of RGB and thermal imaging branches based on these weights. Zhang et al. [15] achieved precise recognition of multi-scale objects by integrating multimodal data and combining it with auxiliary super-resolution (SR) learning techniques. Liu et al. [16] proposed a network combining frequency mining and complementary feature fusion to address the modal discrepancy between RGB and infrared images in object detection. This approach effectively integrates complementary information from both modalities, enhancing detection accuracy and robustness. Zhang et al. [17] focused on the impact of noisy fused feature maps leading to false positives (FP), proposing a novel target-aware fusion strategy (TFDet) for multispectral pedestrian detection. Sun et al. [18] proposed a specificity-guided cross-modal feature reconstruction method (SCFR), which adaptively fuses features from RGB and infrared images, improving target detection accuracy and robustness in low-light and complex background scenarios. To solve the problem of the lack of cross-modal long-range dependency modeling in existing methods, Fang et al. [19] were the first to apply transformers to multimodal object detection, using it to learn long-range dependencies and integrate global contextual information, achieving new heights in multispectral object detection. Shen et al. [20] also applied transformers to the multimodal object detection field, proposing a cross-modal feature enhancement module that enhances single-modal feature representations using global information from complementary modalities. However, these methods struggle to simultaneously capture both excellent global features and local feature details.

## 3. Attention Mechanism

In this section, we introduce the attention mechanisms utilized in this study. Specifically, Section 3.1 presents the CA mechanism, Section 3.2 describes the SE attention mechanism, and Section 3.3 introduces the multi-head self-attention mechanism.

### 3.1. Coordinate Attention

**Figure 1:**
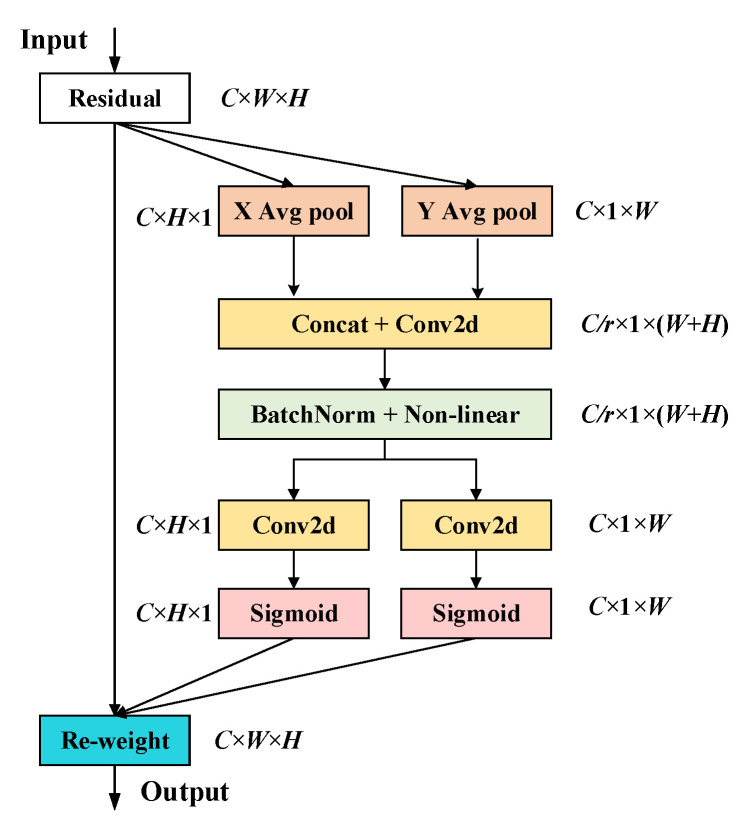
The structure of CA.

The CA (coordinate attention) [21] is a lightweight attention module that effectively combines channel attention with spatial information. It performs global information aggregation separately in the height and width directions, capturing the directional information of the feature map. Especially in complex backgrounds, CA enhances the accuracy of object detection by weighting channels and spatial features. It performs particularly well in complex scenarios. These include background interference, large differences in object scales, and significant aspect ratio variations in image data. Its structure is shown in Figure 1.

In CA, the input feature map with dimensions *C* × *H* × *W* is pooled separately along the X and Y directions, generating feature maps of size *C* × *H* × 1 and *C* × 1 × *W*, respectively. The formulas are as follows:

(1) $$z_{c}^{h}\left(h\right)=\frac{1}{W}\sum_{0\leq i<W}x_{c}\left(h,i\right)$$

(2) $$z_{c}^{w}\left(w\right)=\frac{1}{H}\sum_{0\leq j<H}x_{c}\left(j,w\right)$$

in Equation (1), $x_{c}(h,j)$ represents the c-th channel when the height is h. After being encoded by a convolution kernel of size (1, *W*), the output is $z_{c}^{h}\left(h\right)$. In Equation (2), $x_{c}\left(j,w\right)$ represents the c-th channel when the height is w. After being encoded by a convolution kernel of size (1, *H*), the output is $z_{c}^{w}\left(w\right)$.

Next, the feature maps aggregated along the width and height directions are concatenated along the channel dimension, resulting in a feature map of size *C* × 1 × (*W* + *H*). Then, this feature map undergoes a dimensionality reduction via a shared 1 × 1 convolutional transformation function *F*_1_, which reduces the channel size from *C* to *C*/*r*. Afterward, the reduced feature map is normalized by batch normalization and passed through an activation function, ultimately producing a feature map of size *C*/*r* × 1 × (*W* + *H*) denoted as *f*. The computation formulas are as follows:

(3) $$f=\delta (F_{1\;}([\;z^{h},z^{w}\;]))$$

where δ is the nonlinear activation step. Then, the feature map undergoes 1 × 1 convolution for feature transformation and is passed through a sigmoid activation function to obtain the attention weights in the height direction $g^{h}$ and the width direction $g^{w}$. The computation formulas are as follows:

(4) $$g^{h}=\sigma (F_{h}(\;{f\;}^{h}))$$

(5) $$g^{w}=\sigma (F_{w}({\;f}^{\;w}))$$

in Equations (4) and (5), $f^{\;h}$ and $f^{\;w}$ are the outputs from the previous step, $F_{h}$ and $F_{w}$ represent the corresponding 1 × 1 convolutions, and σ is the sigmoid activation function. Finally, the weighted multiplication is applied to the original feature map to obtain the final output feature. The computation formula is as follows:

(6) $$y_{c}\left(i,j\right)=x_{c}\left(i,j\right)\;\times {\;g}_{c}^{h}\left(i\right)\;\times \;g_{c}^{w}\left(j\right)$$

### 3.2. Squeeze-And-Excitation Network

**Figure 2:**
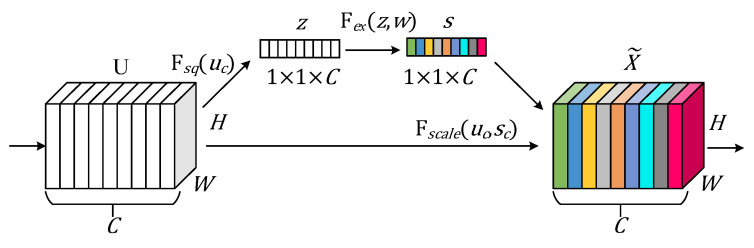
The structure of SE attention.

SENet (squeeze-and-excitation network) [22], proposed by Hu et al., is a novel network architecture designed to adaptively recalibrate channel-wise feature responses. Its core idea is to learn the importance of each feature channel based on the loss during training, thereby enhancing the representation of effective channels while suppressing less relevant ones, ultimately improving model performance. The structure of SENet is shown in Figure 2.

SENet primarily consists of three key steps: squeeze, excitation, and reweighting.

The squeeze operation aggregates feature information across each channel using global average pooling, compressing the channel feature map $u_{c}$ into a scalar $z_{c}$, which quantifies the contribution of each channel to the overall feature representation. The corresponding formula is as follows:

(7) $$z_{c}={\;F}_{\mathrm{sq}}\left(u_{c}\right)=\frac{1}{H\;\times \;W}\sum_{i=1}^{H}\sum_{j=1}^{W}u_{c}\left(i,j\right)$$

where $F_{sq}$ denotes the squeeze operation, and $u_{c}$ represents the feature map of channel c.

The excitation operation inputs the global feature z into a fully connected layer, followed by a nonlinear transformation using the ReLU activation function. Then, the output is passed through another fully connected layer, and the result is mapped to the weight range [0, 1] using a sigmoid activation function. The corresponding formula is as follows:

(8) $$s=F_{\mathrm{ex}}(z,w)=\sigma (w_{2}\cdot \delta (w_{1}\cdot z))$$

where $F_{\mathrm{ex}}$ denotes the excitation operation, σ represents the sigmoid activation function, δ denotes the ReLU activation function, and $w_{1}$ and $w_{2}$ are the weight parameters of the two fully connected layers.

The reweighting operation involves element-wise multiplication of the obtained channel weights $s_{c}$ with the input features $u_{c}$, resulting in the weighted feature map ${\tilde{X}}_{c}$. The corresponding formula is as follows:

(9) $${\tilde{X}}_{c}=F_{\mathrm{scale}}\left(u_{c},s_{c}\right)=s_{c}\cdot u_{c}$$

where $F_{\mathrm{scale}}$ represents the element-wise multiplication of the channel weight $s_{c}$ and the input feature map $u_{c}$ along the channel dimension.

### 3.3. Multi-Head Self-Attention

**Figure 3:**
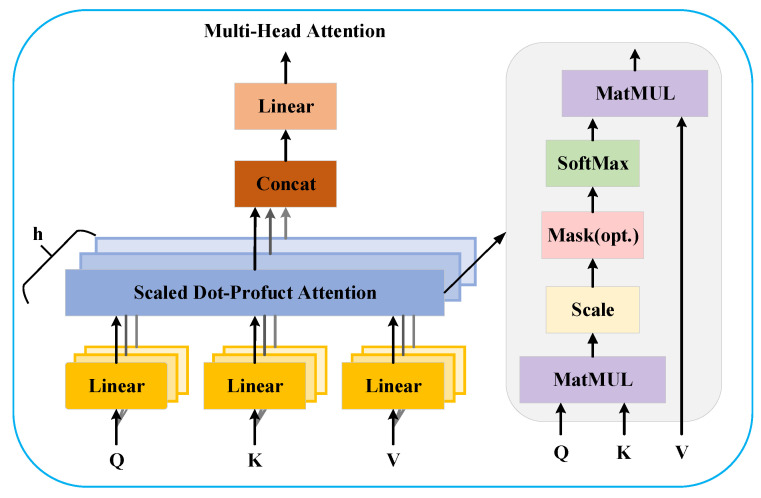
The structure of multi-head self-attention.

MSA (multi-head self-attention) consists of multiple single-head self-attention mechanisms, with each head focusing on different feature dependencies. As illustrated in Figure 3, the inputs to the attention mechanism include the query vector (*Q*), key vector (*K*), and value vector (*V*). Specifically, *Q* represents the query vector for each position in the input sequence, measuring the correlation between the current element and other elements; *K* denotes the key vectors at each position in the input sequence, which together with *Q*, determines the attention weights by computing their similarity; *V* corresponds to the value vectors at each position in the input sequence, containing feature information from the respective positions. The final attention output is obtained as a weighted sum of *V*, computed using the following equation:

(10) $$Q=IW^{Q}$$

(11) $$K=IW^{K}$$

(12) $$V=IW^{V}$$

where I represents the input matrix, while $W^{Q}$, $W^{K}$, and $W^{V}$ denote the weight matrices for the query, key, and value transformations, respectively.

In the multi-head self-attention mechanism, the *Q* is multiplied by the transposed *K^T^* using matrix multiplication (MatMUL) to obtain a similarity matrix, which measures the correlation between elements. Subsequently, a scaling operation is applied to derive the attention matrix, which is then multiplied by the *V* to generate the weighted feature representation. The corresponding mathematical formulation is as follows:

(13) $$Z=\mathrm{Attention}(Q,K,V)\;=\mathrm{softmax}(\frac{QK^{T}}{\sqrt{d_{k}}})V$$

in Equation (13), $1/\sqrt{d_{k}}$ is the scaling factor that prevents the softmax function from entering regions of extremely small gradients when the dot product becomes large, where $d_{k}$ is the dimensionality of the *K*.

(14) $$Z^{\prime }=\mathrm{MultiHead}\left(Q,K,V\right)=\mathrm{Concat}\left(Z_{1},\ldots \ldots ,Z_{h}\right)W^{O}$$

(15) $$Z_{i}=\mathrm{Attention}\left(QW_{i}^{Q},KW_{i}^{K},VW_{i}^{V}\right)$$

in Equation (14), *h* denotes the number of self-attention heads. Among these h self-attention heads, in Equation (15), *i* represents the index of the head, ranging from 1 to *h*.

## 4. Proposed Method

In this section, we first provide an overview of the proposed network architecture. Then, in Section 4.2, we present a detailed explanation of the proposed CGF module, followed by the introduction of the ACF module in Section 4.3.

### 4.1. MFYOLO Structure Overview

**Figure 4:**
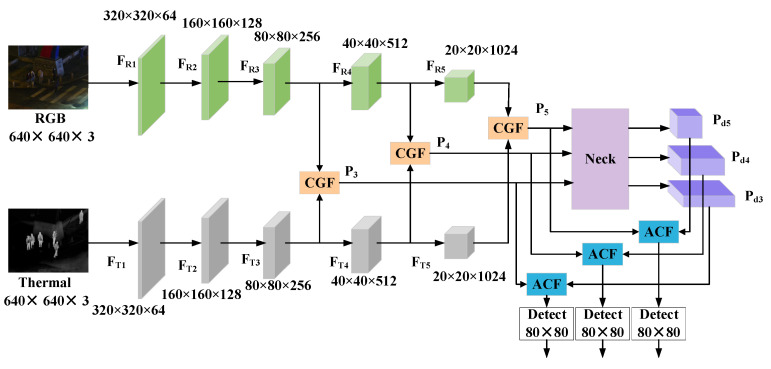
The structure MFYOLO.

MFYOLO is built upon the YOLOv8 framework, extending its feature extraction structure into a dual-stream backbone network, as illustrated in Figure 4. This design enables the model to simultaneously process visible and thermal imaging data, facilitating cross-modal feature interaction and fusion.

During feature extraction, the RGB and thermal inputs pass through a series of convolutional layers and multiple down-sampling operations, generating multi-scale feature maps F_R_ and F_T_, respectively. Among these, feature maps of sizes 80 × 80 × 256, 40 × 40 × 512, and 20 × 20 × 1024 are particularly crucial for object detection. Therefore, we embed the CGF after these feature maps, producing fused feature maps P_3_, P_4_, and P_5_, which are subsequently fed into the neck for further feature fusion and enhancement.

However, deep convolutional layers often lead to the loss of detailed information. To mitigate this issue, we integrate the ACF at the end of the neck in the baseline model. ACF adaptively fuses the shallow features P_3_, P_4_, and P_5_ from the dual-stream backbone with the deep features P_d3_, P_d4_, and P_d5_ obtained through the feature pyramid, thereby effectively calibrating and enhancing the final feature representations fed into the detection head. This process helps in recovering detailed information and improving detection performance.

### 4.2. Content-Guided Fusion Module

In RGB-T object detection tasks, visible and thermal imaging data exhibit strong complementarity, and their effective fusion can significantly enhance model robustness and detection performance under complex conditions. However, most existing transformer-based cross-modal approaches rely heavily on multi-head self-attention to model global contextual information. Such methods often neglect the modeling of local details and spatial structures, which limits the alignment and interaction of fine-grained semantic information across modalities. Furthermore, although some studies have attempted to combine convolutional networks with transformers, the majority adopt simple module stacking strategies without establishing a synergistic optimization mechanism between global and local representations. As a result, these methods lack both architectural coherence and innovative fusion strategies.

**Figure 5:**
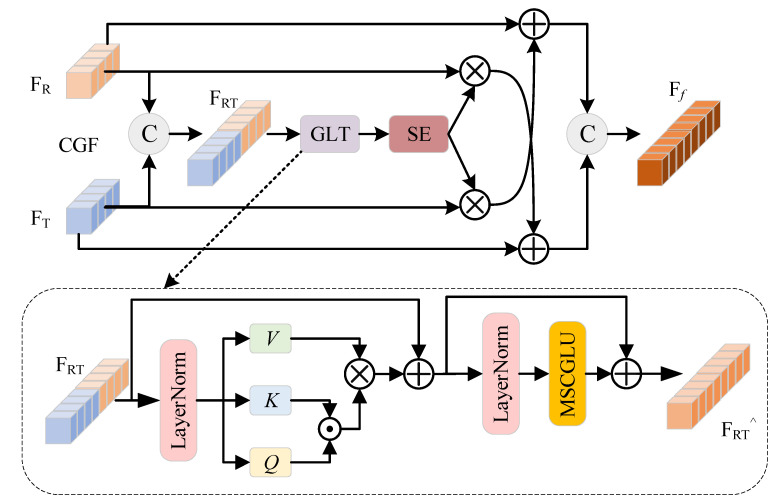
The structure of CGF and GLT.

To address this issue, we proposed CGF, as illustrated in Figure 5. The core innovation of CGF lies in the design of a “globally-local guided” cross-modal fusion structure, which breaks through the traditional paradigm of merely stacking convolutional and transformer modules in parallel. Specifically, CGF first concatenates the features from visible and thermal modalities along the channel dimension to form a unified representation. This representation is then fed into a global–local transformer (GLT) for joint modeling. The GLT incorporates a multi-head self-attention mechanism (MSA) to capture global semantic dependencies, and our proposed multi-scale convolutional gated linear unit (MSCGLU) to enhance local feature representation. MSCGLU integrates multi-scale depthwise separable convolutions with a gated mechanism and introduces a coordinate attention component to enable dynamic feature adjustment with spatial awareness.

Subsequently, the features processed by the GLT are fed into the SE attention mechanism, which adaptively adjusts channel-wise attention weights to enhance the representation of crucial feature channels. Finally, the fused features are split into two modality-specific branches, and each is element-wise weighted and residually added to the corresponding original modality features, thereby achieving semantically guided complementary fusion between modalities.

**Figure 6:**
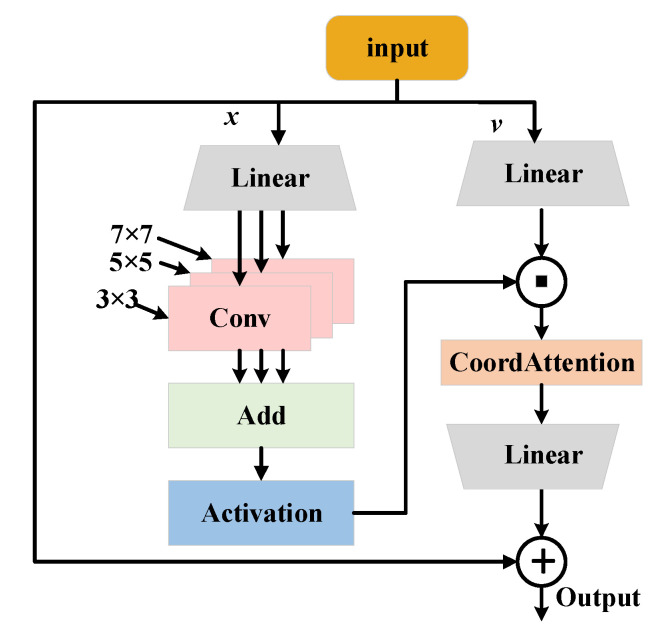
The structure of MSCGLU.

Traditional transformers extract global information using multi-head attention and perform feature transformation and reshaping via the MLP (multi-layer perceptron). However, since MLP consists of stacked fully connected layers, it lacks the ability to model local features effectively. To address this limitation, we replace the MLP with the MSCGLU to enhance the collaborative modeling of global and local features, thereby improving fine-grained feature representation. MSCGLU’s structure is shown in Figure 6.

The key innovation of GLT lies in replacing the MLP layer in the classic transformer with MSCGLU. Compared to traditional fully connected MLP layers, MSCGLU has stronger local sensing ability.

MSCGLU inherits the core gating mechanism of GLU [23,24], enabling selective information flow through feature segmentation and dynamic weighting, thereby enhancing the network’s adaptive learning capability. Building on this foundation, the module incorporates multi-scale convolution and CA mechanisms and achieves deep integration of the three through structural design.

Specifically, after the input features are divided into the main branch and the gating branch, the main branch sequentially processes the input through convolutional kernels of 3 × 3, 5 × 5, and 7 × 7 receptive fields, allowing it to extract multi-scale local representations. This significantly improves the model’s ability to capture objects of varying sizes and model complex spatial structures. Importantly, this multi-scale structure is not applied in isolation. Instead, it is coupled with the GLU structure via a shared gating branch, enabling the gated modulation of multi-scale information before fusion. This design enhances the spatial adaptability of the selective response mechanism.

To further improve the modeling of spatial saliency, the MSCGLU module introduces the CA mechanism after the multi-scale convolution in the main branch. Unlike a simple stack of independent modules, CA is employed as an attention enhancement component guided by the preceding multi-scale features. After being processed by multi-scale convolution, the features have already acquired rich spatial structural representations. The CA module then exploits its directional global modeling capability (aggregating information along horizontal and vertical axes) to extract the most salient spatial regions. Notably, CA does not apply generic reweighting to the raw features. Instead, it leverages the spatial priors provided by multi-scale convolution to refine and focus the spatial response map, achieving scale-guided spatial attention modulation. This integrative design empowers MSCGLU with robust feature representation capabilities across both scale and spatial dimensions, facilitating more accurate and discriminative downstream detection performance. The specific steps are as follows:

MSCGLU first splits the input along the channel dimension into *x* and *v*. The feature *x* is then processed through multi-scale convolutions, formulated as follows:

(16) $$x_{\mathrm{multi}}=\sum_{i\in \left\{3,5,7\right\}}{w_{i\;}}^{*}\;({\mathrm{FC}}_{1}(x))$$

here, * denotes the convolution operation, $w_{i}$ represents the convolution kernel with a size of i × i, and FC1 is a fully connected layer.

Next, the multi-scale features, after being processed by an activation function, undergo element-wise multiplication with the gating signal *v*. Finally, spatial information is supplemented through CA, and the resulting features are summed with the original input *x* to obtain the final output.

(17) $$y={\;\mathrm{FC}}_{2}(CA(\mathrm{GELU}\left(x_{\mathrm{multi}}\right)\cdot {\mathrm{FC}}_{1}(v)))+x$$

where GELU serves as the activation function, and FC2 is a fully connected layer.

### 4.3. Adaptive Calibration Fusion Module

**Figure 7:**
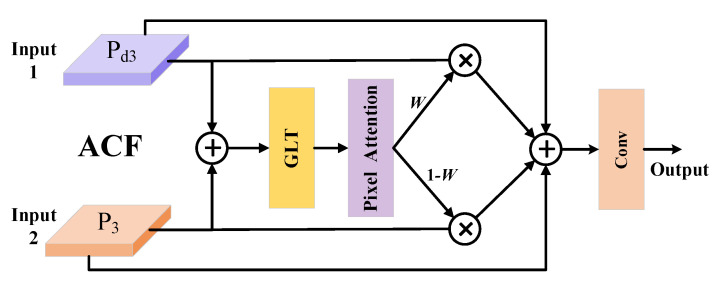
The structure of ACF.

During the deep convolution process, input features may gradually lose fine-grained information, and this phenomenon is particularly detrimental in the RGB-T feature fusion process. To alleviate this issue, we draw on the design ideas from DEA-Net [25] and proposed ACF, as shown in Figure 7.

Taking the shallow feature P_3_ and deep feature P_d3_ as examples, we first perform a simple addition of the two as the starting point for initial fusion. Then, this initial fused feature is fed into the GLT module to extract the global and local contextual information, resulting in a guiding feature. Next, the module compares the initial fused feature with the guiding feature to generate a spatial attention map *W.* After applying the sigmoid activation, the values of W range between [0, 1], reflecting the model’s preference for relying on one of the input features at each spatial position. Finally, the module uses *W* to weight P_3_, while using (1 − *W*) to weight P_d3_, hereby achieving adaptive fusion of both features along the spatial dimension. The core of this mechanism is to guide the model in dynamically adjusting the fusion ratio between shallow and deep features based on feature differences at different spatial positions, thus improving the overall fusion performance.

## 5. Experiments and Results

In this section, we first introduce the experimental environment and key training parameters in Section 5.1. Then, in Section 5.2, we describe the datasets used in this study. Next, Section 5.3 presents the evaluation metrics employed for experimental analysis. Finally, Section 5.4 and 5.5 provide the ablation study and comparative experiment analysis, respectively.

### 5.1. Experiments Setup

We implemented the proposed method using PyTorch 1.12.1 and CUDA 11.2 and conducted training and testing on a workstation equipped with an RTX A6000 GPU. The detailed software and hardware configurations for the experiments are provided in Table 1.

To ensure fair comparisons between models and achieve optimal training performance, we fine-tuned the experimental parameters through extensive trials. The final configuration is as follows: the input image size for both modalities is fixed at 640 × 640, and a batch of 8 images is randomly sampled per iteration. The training is conducted for 200 epochs. Data augmentation is employed to enhance input diversity and accelerate model convergence. The entire network is optimized using SGD for a sufficient number of epochs, with an initial learning rate set to 0.01, weight decay set to 0.0005, and momentum set to 0.937. The IoU threshold is set to 0.7.

### 5.2. Dataset

In this study, we utilized publicly available, pre-aligned visible and thermal imaging datasets, LLVIP [26] and M3FD [27].

LLVIP: The LLVIP dataset is a visible-infrared paired pedestrian dataset designed for low-light vision research. It contains 30,976 images, forming 15,488 pairs, captured across 24 nighttime scenes and 2 daytime scenes.

M3FD: The M3FD dataset is a benchmark dataset for multispectral object detection, covering complex road scenarios such as daytime, cloudy, and nighttime conditions. It provides 4200 pairs of aligned visible-infrared images with annotations for six object categories: pedestrian, car, bus, motorcycle, truck, and street lamp.

### 5.3. Evaluation Indicators

We divide the evaluation of the algorithm into subjective and objective evaluation metrics. Subjective evaluation involves visually inspecting the detection results of different algorithms to assess their performance and identifying any false detections or missed detections. Objective evaluation quantifies performance using miss rate (MR) and mean average precision (mAP) as evaluation metrics. The formulas for recall and mAP are given as follows:

(18) $$R=\frac{TP}{TP+FN}$$

(19) $$P=\frac{TP}{TP+FP}$$

(20) MR=1−R

(21) $$\mathrm{mAP}=\frac{1}{n}\sum_{i=0}^{n}{\mathrm{AP}}_{i}$$

(22) $$\mathrm{AP}=\int_{0}^{1}P(R)dR$$

in Equations (18) and (19), TP denotes the number of positive samples correctly predicted as positive, FP refers to the number of negative samples incorrectly predicted as positive, and FN represents the number of positive samples incorrectly predicted as negative. In Equation (21), *n* represents the total number of categories. Typically, mAP50 is used to represent the mean AP across all categories at an IoU threshold of 0.5, while mAP95 represents the mean AP across all categories at an IoU threshold of 0.95.

### 5.4. Ablation Experiment

#### 5.4.1. Cumulative Benefit Analysis of the CGF Module

To verify the effectiveness of the CGF module in multimodal object detection tasks, we conducted an ablation study on the LLVIP dataset, specifically analyzing the impact of different numbers of CGF modules on detection performance. The experimental results are presented in Table 2.

As shown in the data, with an increasing number of CGF modules, mAP95 consistently improves, rising from 61.4% to 63.7%, an increase of 2.3% percentage points. This indicates that the CGF module effectively enhances feature extraction and fusion, thereby improving object detection performance. Meanwhile, mAP50 also exhibits a stable upward trend, reaching 95.7% with two CGF modules and further increasing to 96.1% with three CGF modules.

Notably, the improvement in mAP95 is more significant, suggesting that the CGF module maintains robust detection capability even under stricter IoU threshold conditions. This further demonstrates that the CGF module effectively optimizes cross-modal information interaction, enhancing the robustness and generalization ability of the detection model. Therefore, the experimental results fully validate the effectiveness of the CGF module and indicate its strong scalability in multimodal object detection tasks.

#### 5.4.2. Ablation Study on Algorithm

In this study, we incorporate CGF and ACF into the dual-backbone YOLOv8 (Baseline) to develop the final MFYOLO model. To validate the effectiveness of the proposed improvements, we incrementally add each module to the baseline and conduct a comparative evaluation of the final result.

To better distinguish the performance gains from parameter expansion and those from module design and to verify the superiority of the ACF module over residual connections and Swin transformer, we introduce three comparison models in Table 3: Baseline_Big (increased width and depth to 6.7 M parameters), Baseline + Swin (replacing ACF with Swin transformer), and Baseline + CGF + Res (replacing ACF with residual connections).

From Table 3, we can see that each stage of the model achieves performance improvements across various metrics on both the LLVIP and M3FD datasets.

On the LLVIP dataset, adding the CGF module reduces the MR by 1.0% compared to the baseline, while mAP50 and mAP95 increase by 0.8% and 2.3%, respectively. With the additional incorporation of the ACF module, MFYOLO achieves a 1.7% lower MR than the baseline, along with 1.1% and 2.4% improvements in mAP50 and mAP95, respectively.

On the M3FD dataset, integrating CGF reduces the MR by 1.1% relative to the baseline, while mAP50 and mAP95 improve by 1.6% and 1.0%, respectively. Further adding the ACF module results in a 3.9% reduction in MR and increases mAP50 and mAP95 by 4.5% and 3.9%, respectively, highlighting the significance of ACF in detection tasks under complex environments.

Moreover, compared to the Baseline_Big model with similar parameters, the proposed model outperforms it on both datasets, indicating that the performance improvement is not solely due to an increase in parameter size. When comparing Baseline + Swin with Baseline + CGF, although the former has 11.1 M parameters and an increase of 5.1 M, its performance in MR and mAP50 is similar to Baseline + CGF. However, Baseline + Swin shows a decrease of 2.5% and 4.4% in mAP95 on the LLVIP and M3FD datasets, respectively, demonstrating that CGF is more advantageous than Swin Transformer in cross-modal detection tasks. Lastly, when replacing the ACF module with a residual connection, the model’s parameter count increases to 7.4 M, but its performance in MR, mAP50, and mAP95 is worse than that of Baseline + CGF + ACF, further confirming that the benefits brought by ACF cannot be achieved through simple residual connections.

Overall, these experimental results on both datasets demonstrate the effectiveness of the proposed CGF and ACF modules. The two modules facilitate the efficient fusion of RGB and thermal-based features while improving information calibration within deep feature representations.

#### 5.4.3. Efficiency Analysis

As shown in Table 4, YOLOv8 achieves the fastest inference time of 8.9 ms among all models. With the introduction of a dual-backbone architecture, the inference time of the baseline increases to 9.2 ms. The proposed MFYOLO model, incorporating both CGF and ACF modules, further increases the inference time to 11.7 ms. When the ACF module is replaced with a residual connection, the inference time of Baseline + CGF + Res reaches 12.2 ms. Notably, Baseline + CGF + Res has both a larger parameter size and longer inference time than MFYOLO, yet it delivers inferior detection accuracy. Furthermore, although Baseline_Big shares the same number of parameters as MFYOLO and demonstrates slightly faster inference, its detection performance is significantly worse.

Overall, although MFYOLO incurs an additional 2.8 ms in inference time compared to YOLOv8, it achieves significantly higher detection accuracy. Moreover, the 11.7 ms inference time is sufficient to meet the real-time requirements of most practical object detection tasks. Therefore, the structural enhancements introduced in MFYOLO demonstrate clear practical value in terms of the trade-off between accuracy and efficiency.

### 5.5. Comparison with Other Algorithms

#### 5.5.1. Quantitative Analysis

On the LLVIP dataset, Table 5 presents the detection performance of MFYOLO compared to other networks on the LLVIP dataset. It can be observed that the mAP50 and mAP95 of our model are 96.4% and 63.8%, surpassing other single-modal networks. Even compared to dual-modal transformer-based networks such as CFT and ICAFusion, MFYOLO consistently outperforms them across all metrics. This demonstrates that, with the combined effect of the CGF and ACF modules, MFYOLO achieves more accurate detection.

Specifically, CFT employs a transformer-based self-attention mechanism to jointly model intra- and inter-modality relationships, enhancing the global representation of multispectral features. ICAFusion, on the other hand, introduces iterative cross-attention to establish fine-grained interactions between modalities and improves fusion efficiency through parameter sharing. While both methods offer innovations in multimodal fusion, they often overlook precise local detail modeling or the coordination between global and local features.

In contrast, MFYOLO integrates our proposed CGF (content-guided fusion) and ACF (adaptive calibration fusion) modules to effectively combine global semantics with local structural information. This enables dynamic alignment and enhancement of cross-modal features, resulting in superior detection accuracy and robustness.

On the M3FD dataset, Table 6 presents the detection performance of MFYOLO compared to other networks on the M3FD dataset. Similarly, it can be observed that the mAP50 and mAP95 of our model are 84.2% and 56.6%, outperforming other single-modal networks as well as the mainstream models TarDAL and CDDFuse [28] on the M3FD dataset. This further demonstrates that our approach achieves higher accuracy in cross-modal object detection.

TarDAL employs a dual-adversarial learning strategy to achieve cross-modality feature alignment, enhancing detection robustness across diverse scenarios. However, its ability to model local details remains limited. CDDFuse, on the other hand, focuses more on multimodal image fusion, utilizing correlation-driven dual-branch feature decomposition to improve fusion quality. While effective for image generation tasks, it still lacks feature selectivity and precise target localization in detection tasks. In contrast, the proposed MFYOLO is specifically tailored for object detection. By integrating the CGF module and the ACF module, MFYOLO enables dynamic guidance and alignment of cross-modal features while enhancing joint modeling of global semantics and local structures. As a result, MFYOLO achieves higher accuracy in cross-modality object detection tasks.

#### 5.5.2. Qualitative Analysis

**Figure 8:**
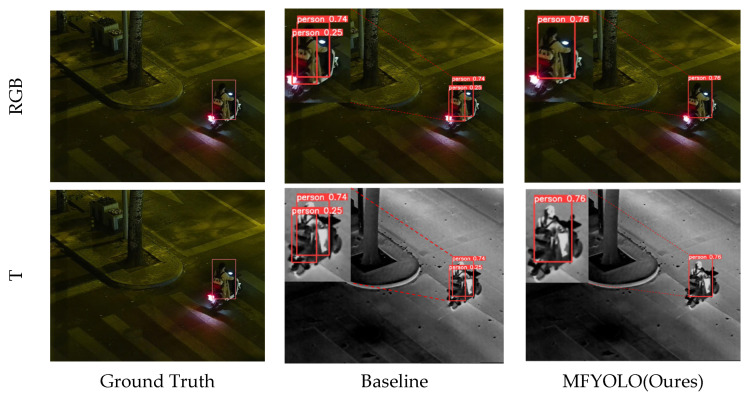
Comparison of detection performance on the LLVIP.

**Figure 9:**
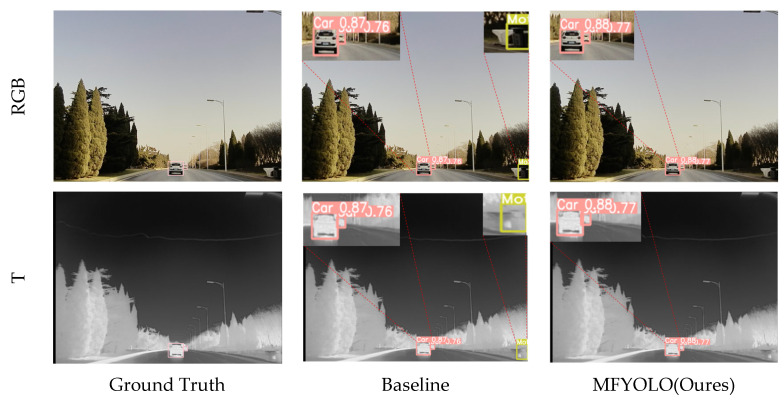
Comparison of detection performance on the M3FD.

In order to make a more intuitive evaluation of the detection results, we qualitatively compare the proposed method with the baseline on the LLVIP and M3FD datasets, as shown in Figure 8 and Figure 9. Upon examining Figure 8, we observe that when there is only one target in the image, MFYOLO correctly detects it, while the baseline model mistakenly recognizes the target’s backpack as a person, resulting in a false positive detection.

The first column of Figure 8 presents the original image, where a single target is annotated. The second column shows the detection results of the baseline model, while the third column displays the results of MFYOLO. The baseline model incorrectly detects the target’s backpack as a person, whereas MFYOLO accurately identifies the target without misclassification. This demonstrates that MFYOLO achieves superior detection performance in low-light environments.

The first column of Figure 9 presents the original image, where two targets are annotated. The second column displays the detection results of the baseline model, while the third column shows the results of MFYOLO. Although the baseline model successfully detects all targets, it misclassifies the trash bin in the lower right corner as a motorcycle. In contrast, MFYOLO correctly identifies all objects without errors. This demonstrates that MFYOLO achieves superior detection performance even in daytime scenarios with small targets.

## 6. Discussion

This study addresses two key challenges in multimodal object detection: (1) the difficulty of balancing local and global features during transformer-based feature fusion, and (2) the degradation of features during multiple convolutional operations. We propose the CGF, which embeds convolutional operations within the transformer architecture to enable the collaborative capture of both global and local features. The ACF utilizes shallow features to adaptively calibrate and fuse deep features, thereby enhancing fine-grained details.

Although CGF and ACF significantly improve performance, there remains room for further improvement in modality fusion. We initially attempted to use convolutional attention to capture global information, but this approach did not meet expectations. Ultimately, we adopted the transformer’s multi-head attention mechanism and combined it with MSCGLU to enhance local information. While this method improved performance, it also increased the complexity of the network.

Based on the parameter analysis in Table 2, we found that adding CGF increased the network’s parameter count by 1.6 M, and the addition of ACF further increased it to 6.7 M. This increase in parameters is not conducive to practical applications. Therefore, future work will focus on optimizing the model architecture to improve both accuracy and efficiency.

## 7. Conclusions

This paper builds upon previous research and focuses on addressing two key challenges: the limitations of traditional transformer models in local attention capabilities and the degradation of deep features during multiple convolution operations, which leads to the loss of fine-grained details. To address these issues, we propose MFYOLO, a dual-backbone cross-modal object detection model based on YOLOv8n. This model integrates the content-guided fusion (CGF) module and the adaptive calibration fusion (ACF) module, both of which aim to significantly enhance the performance of multimodal object detection.

Specifically, the CGF module introduces a multi-head attention mechanism to capture global information, while MSCGLU is used to enhance local information. This dual approach ensures a balanced extraction of both global and local features. By splitting and weightedly combining feature maps, the CGF module improves the fusion of detailed features between different modalities. The final output is a fused feature map with bidirectional information guidance.

The ACF module combines shallow and deep features, supplementing deep features with detailed information. This effectively mitigates the problem of feature degradation. Overall, our proposed method not only compensates for the limitations of traditional transformers in local perception but also alleviates feature degradation resulting from multiple convolution operations.

We conducted experiments on two RGB-T datasets. The results show that our method outperforms other state-of-the-art multimodal detection approaches, both in terms of detection accuracy and overall performance.

## Figures and Tables

**Table 1:** List of hyperparameters.

Hyperparameters	Value
Optimizer	SGD
Learning rate	0.01
Momentum	0.937
Input shape	640 × 640
Weight decay	0.0005
Training epochs	200
IoU threshold	0.7

**Table 2:** Ablation study results of the CGF module.

Number (CGF)	mAP50 (%)	mAP95 (%)
0	95.3	61.4
1	94.8 (−0.5)	62.4 (+1.0)
2	95.7 (+0.4)	63.0 (+1.6)
3	96.1 (+0.8)	63.7 (+2.3)

**Table 3:** The results of ablation experiment results. (A lower MR indicates better performance, while higher mAP50 and mAP95 values are preferred).

Methods	Input	Params (M)	MR (%)		mAP50 (%)		mAP95 (%)	
			LLVIP	M3FD	LLVIP	M3FD	LLVIP	M3FD
YOLOv8	RGB	3.0	18.0	28.0	88.0	80.5	49.2	52.4
YOLOv8	T	3.0	12.2	29.6	94.5	77.4	61.5	50.8
Baseline	RGB + T	4.4	10.7	26.7	95.3	79.7	61.4	52.7
Baseline_Big	RGB + T	6.7	11.5	25.5	95.6	80.6	62.6	53.4
Baseline + CGF	RGB + T	6.0	9.7	25.6	96.1	81.3	63.7	53.7
Baseline + Swin	RGB + T	**11.1**	10.2	25.1	96.2	81.6	61.2	49.3
Baseline + CGF + Res	RGB + T	7.4	12.7	25.3	95.3	82.7	61.4	55.9
Baseline + CGF + ACF	RGB + T	6.7	**9.0**	**22.8**	**96.4**	**84.2**	**63.8**	**56.6**

**Table 4:** Inference time comparison.

Methods	Params (M)	Inference (ms)
YOLOv8	**3.0**	**8.9**
Baseline	4.4	9.2
Baseline_Big	6.7	10.1
MFYOLO	6.7	11.7
Baseline + CGF + Res	7.4	12.2

**Table 5:** Comparison with other algorithms on the LLVIP.

Method	Input	mAP50 (%)	mAP95 (%)
YOLOv5	RGB	85.7	48.1
YOLOv5	T	93.6	59.6
YOLOv6	RGB	85.6	49.3
YOLOv6	T	94.4	62.4
YOLOv8	RGB	88.0	49.2
YOLOv8	T	94.5	61.5
ICAFusion	RGB + T	95.8	59.0
CFT	RGB + T	95.8	62.3
Baseline	RGB + T	95.3	61.4
MFYOLO (Ours)	RGB + T	**96.4**	**63.8**

**Table 6:** Comparison with other algorithms on the M3FD.

Method	Input	mAP50 (%)	mAP95 (%)
YOLOv5	RGB	65.6	42.2
YOLOv5	T	64.7	41.8
YOLOv6	RGB	75.6	49.0
YOLOv6	T	73.8	47.8
YOLOv8	RGB	80.5	52.4
YOLOv8	T	77.4	50.8
TarDAL	RGB + T	81.9	-
CDDFuse	RGB + T	82.0	-
Baseline	RGB + T	79.7	52.7
MFYOLO (Ours)	RGB + T	**84.2**	**56.6**

## Data Availability

The dataset used in this study can be accessed at the following link. LLVIP: https://github.com/bupt-ai-cz/LLVIP/blob/main/download_dataset.md (accessed on 5 July 2023) M3FD: https://github.com/JinyuanLiu-CV/TarDAL (accessed on 12 July 2023).

## Abbreviations

The following abbreviations are used in this manuscript:

YOLO	You Only Look Once
MFYOLO	Multimodal Fusion YOLO
CNN	Convolutional neural networks
RCNN	Region-based convolutional neural network
SSD	Shot multi-box detector
CA	Coordinate attention
SE	Squeeze-and-excitation network
MSA	Multi-head self-attention
CGF	Content-guided fusion
GLT	Global–local transformer
GLU	Gated linear unit
MSCGLU	Multi-scale convolutional gated linear unit
ACF	Adaptive calibration fusion
SGD	Stochastic gradient descent
mAPx	mean average precision while confidence at 0.x
MR	Miss rate
IOU	Intersection of union
